# Perilipin 5 deficiency aggravates cardiac hypertrophy by stimulating lactate production in leptin-deficient mice

**DOI:** 10.1186/s13062-023-00411-8

**Published:** 2023-09-04

**Authors:** Lele Jian, Xing Gao, Chao Wang, Xiao Sun, Yuqiao Xu, Ruili Han, Yuying Wang, Shenhui Xu, Lan Ding, Jingjun Zhou, Yu Gu, Yuanlin Zhao, Ying Yang, Yuan Yuan, Jing Ye, Lijun Zhang

**Affiliations:** 1 Department of Clinical Diagnosis, Tangdu Hospital, Fourth Military Medical University, Xi’an, 710038 China; 2 Shaanxi Provincial Corps, Chinese People’s Armed Police Force, Xi’an, 710054 China; 3 State Key Laboratory of Cancer Biology, Department of Pathology, Xijing Hospital and School of Basic Medicine, Fourth Military Medical University, Xi’an, 710032 China; 4 Department of Pathology, The General Hospital of Western Theater Command, Chengdu, 610083 China; 5 Department of CardiologyXijing Hospital, Fourth Military Medical University, Xi’an, 710032 China; 6 Department of Physiology and Pathophysiology, School of Basic Medicine, Fourth Military Medical University, Xi’an, 710032 China

**Keywords:** Perilipin 5, Glucose utilization, Insulin resistance, Lactate, Myocardial hypertrophy

## Abstract

**Background:**

Perilipin 5 (Plin5) is well known to maintain the stability of intracellular lipid droplets (LDs) and regulate fatty acid metabolism in oxidative tissues. It is highly expressed in the heart, but its roles have yet to be fully elucidated.

**Methods:**

Plin5-deficient mice and Plin5/leptin-double-knockout mice were produced, and their histological structures and myocardial functions were observed. Critical proteins related to fatty acid and glucose metabolism were measured in heart tissues, neonatal mouse cardiomyocytes and Plin5-overexpressing H9C2 cells. 2-NBDG was employed to detect glucose uptake. The mitochondria and lipid contents were observed by MitoTracker and BODIPY 493/503 staining in neonatal mouse cardiomyocytes.

**Results:**

Plin5 deficiency impaired glucose utilization and caused insulin resistance in mouse cardiomyocytes, particularly in the presence of fatty acids (FAs). Additionally, Plin5 deficiency increased the NADH content and elevated the expression of lactate dehydrogenase (LDHA) in cardiomyocytes, which resulted in increased lactate production. Moreover, when fatty acid oxidation was blocked by etomoxir or LDHA was inhibited by GSK2837808A in Plin5-deficient cardiomyocytes, glucose utilization was improved. Leptin-deficient mice exhibited myocardial hypertrophy, insulin resistance and altered substrate utilization, and Plin5 deficiency exacerbated myocardial hypertrophy in leptin-deficient mice.

**Conclusion:**

Our results demonstrated that Plin5 plays a critical role in coordinating fatty acid and glucose oxidation in cardiomyocytes, providing a potential target for the treatment of metabolic disorders in the heart.

**Graphic abstract:**

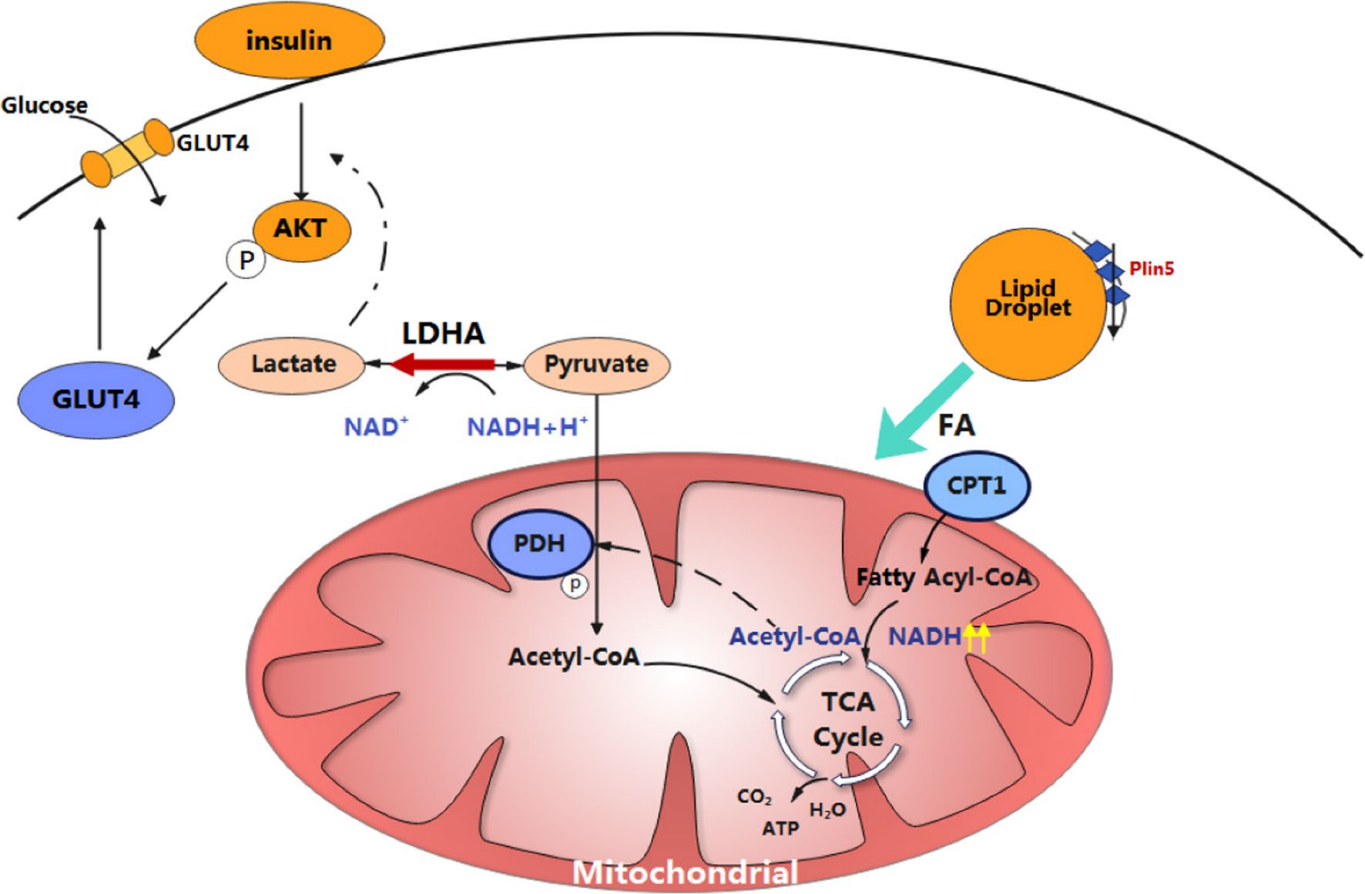

## Introduction

Incessant production of energy in the heart is vital for the heart to maintain its continuous pumping function to guarantee adequate oxygen and nutrient supply to the body. To meet the high energetic demand, the heart possesses high flexibility in the use of any kind of substrate, including fatty acids, glucose, lactate, and, to a lesser extent, ketone bodies and amino acids [1]. Glycolysis is a major source of energy for proliferating cardiomyocytes during early cardiac development, but fatty acid β-oxidation (FAO) becomes the main source of energy for the adult heart with its increased mitochondrial oxidative capacity [2]. Under basal conditions, almost 50%-70% of the energy in the adult heart is derived from FAO [3–5]. The reprogramming of myocardial substrate utilization is closely related to cardiac pathology. For example, fatty acid utilization is further increased in diabetic hearts [6, 7], while energy metabolism switches to a more foetal phenotype in hypertrophic cardiomyopathy [8]. Thus, it is critical to recognize the modulation of cardiac substrate utilization.

Lipid droplets (LDs) are ubiquitous cellular organelles that store excess neutral lipids. There are only a few small LDs in cardiomyocytes, but the rapid turnover of cardiac LDs indicates their vital roles in modulating fatty acid metabolism [9, 10]. Perilipin 5 (Plin5) is a perilipin family member expressed in highly oxidative tissues, such as heart, liver, skeletal muscle, and brown adipose tissue (BAT) [11]. It plays a very important role in maintaining the stability of intracellular LDs and regulating fatty acid metabolism [12]. Recently, it was reported that Plin5 deficiency can induce insulin resistance in the skeletal muscle and liver [13, 14]. However, its effect on myocardial substrate metabolism is poorly understood and awaits further clarification.

In this study, glucose metabolism and insulin signalling were investigated in Plin5-knockout mice and cardiomyocytes. We also explored the roles and mechanism of Plin5 deficiency in the development of myocardial hypertrophy in leptin-deficient mice, which are characterized by abnormal lipid metabolism. This study demonstrates that myocardial Plin5 plays crucial roles in maintaining the homeostasis of glucose and fatty acid oxidation, which provides an experimental basis for preventing myocardial hypertrophy in metabolic disorders.

## Material and methods

### Mice

Wild-type, *Plin5*^−/−^/*leptin*^+/+^ (Plin5-KO), *Plin5*^+/+^/*leptin*^−/−^ (leptin-KO), and *Plin5*^−/−^/*leptin*^−/−^ (DKO) mice (backcrossed onto C57BL/6) were used in this study. All experimental mice were generated by *Leptin*^±^ and *Plin5*^±^ replication. The mice were housed in the SPF-class housing of the laboratory (12-h light/12-h dark cycle) with ad libitum access to water and a chow diet. All experimental mice were 12 weeks old unless otherwise specified, and the animal studies were approved by the animal ethics committee of the Fourth Military Medical University.

### Oil Red O staining

Twelve-week-old mice were sacrificed, and the hearts were immediately removed and placed into isopentane. The hearts were frozen at − 20 °C and sliced into 10-μm sections. The sections were fixed in 4% paraformaldehyde for 20 min. Then, the sections were washed with 60% (v/v) isopropanol for 2 min, stained with 0.3% (w/v) Oil Red O solution in 60% isopropanol for 6 h, washed with 60% isopropanol again and subsequently with PBS, and then counterstained with haematoxylin for 2 min. They were observed and imaged under a microscope (Olympus BX50). Then, the Oil Red O dye was solubilized in 100% isopropanol and quantified by measuring absorbance at 520 nm using a microplate spectrophotometer (Thermo Scientific Multiskan Go).

### Cardiomyocyte hypertrophy assessments

To measure the cardiomyocyte size, the hearts were cut transversally, fixed in 4% paraformaldehyde overnight, dehydrated in a graded isopropanol series, and embedded in paraffin. Serial sections of 5 µm thickness were prepared, stained with haematoxylin and eosin (H&E), and then examined under a light microscope (Olympus BX53). Five fields were randomly imaged at a magnification of × 400. The sizes of the cells were measured using Olympus cellSens™ Entry image analysis software.

### Electron microscopy

Dissected heart tissues were fixed with 2.5% cold glutaraldehyde overnight at 4 °C. After washing with PBS, the tissues were further fixed with 1% osmium tetroxide for 1.5 h. The specimens were dehydrated in a series of graded acetone solutions and embedded in Epon 812 resin. Ultrathin section (70 nm) were cut, placed onto slides and counterstained with uranyl acetate and lead citrate for HT-7800 transmission electron microscope observation.

### Generation of Plin5-overexpressing H9C2 cells

H9C2 cardiomyocyte cells were cultured in Dulbecco’s modified Eagle’s medium (DMEM) (Gibco) supplemented with 10% FBS at 37 °C in a 5% CO_2_ humidified atmosphere. For lentivirus production, 293 T cells were cultured in DMEM supplemented with 10% FBS in 6 cm dishes when 60–70% confluence was reached. The plasmids pLenti-CMV-mPlin5, pAX2 and pVSVG (5:3:2) were diluted in 200 μl of serum-free medium and transfected into 293 T cells by PEI. pLenti-CMV-empty (Origene, ps100069) was used as an empty vehicle control. The culture medium was changed 16 h later, and virus was collected 72 h later to infect the H9C2 cardiomyocytes. H9C2 cells infected with pLenti-CMV-empty (EV) and pLenti-CMV-Plin5 (Plin5-OE) were screened with 2 μg/ml puromycin. Unless otherwise specified, the disposal of fatty acids used in the experiment refers to 100 μM oleic acid (OA, Sigma‒Aldrich) and 100 μM palmitic acid (PA, Sigma‒Aldrich).

### Isolation and culture of neonatal mouse cardiomyocytes

Whole mouse neonatal hearts at Day 1 were quickly dissected, washed with cold PBS and then minced and digested with 5 ml of collagenase I (1 mg/ml) for 8 min in a cell incubator. The supernatant was collected into DMEM with 20% FBS. The digestion procedure was repeated 8 ~ 10 times. The supernatant was centrifuged at 800 rpm for 8 min, and the sediment was resuspended in DMEM with 20% FBS when the tissue was completely digested. The resuspended cells were seeded onto 10 cm dishes for 2 h at 37 °C to remove fibroblasts and then seeded onto 6 cm plastic dishes at an appropriate density.

### Immunofluorescence

For fluorescent immunostaining, H9C2 and neonatal mouse cardiomyocytes were cultured in 12-well plates with coverslips. After treatment with 100 μM OA and 100 μM PA for 24 h, the cells were fixed with 4% paraformaldehyde and permeabilized with 0.1% Triton X-100 for 10 min. After being blocked with 10% normal goat serum in PBS, the cells were incubated with a rabbit primary antibody against GLUT4 (1:200) (Abclonal) overnight at 4 °C. This step was followed by incubation with a fluorochrome-conjugated secondary donkey anti-rabbit antibody for 1 h in the dark (Invitrogen). Hoechst 33258 (Sigma) was used to counterstain the nuclei. The cells were visualized and photographed with a fluorescence microscope (Olympus IX83). The relative quantification of GLUT4 fluorescence intensity in the cell membrane was calculated by Image-Pro Plus 6.0 (Media Cybernetic Inc., USA) image analysis software.

### MitoTracker and BODIPY 493/503 staining

Neonatal mouse cardiomyocytes were washed with 1 ml of PBS to remove the medium. The cells were incubated with 200 nM MitoTracker (Sigma) stain for 20 min in the dark at room temperature. The cells were washed with 1 ml of PBS three times. The cells were fixed with 4% paraformaldehyde for 20 min. Then, the cells were incubated with 2 μM BODIPY 493/503 (Sigma) staining solution in the dark for 20 min at 37 °C. The cells were washed using 1 ml of PBS to remove the staining solution. Finally, the nuclei were stained with Hoechst 33258 (Sigma) for 5 min. The cells were observed and photographed by fluorescence microscopy (Olympus IX83). Quantification analysis of lipid contents and mitochondria was performed using Image-Pro Plus 6.0 (Media Cybernetic Inc., USA) image analysis software.

### 2-NBDG assay

Cells were plated at 1 × 10^4^/well in black 96-well plates in DMEM supplemented with 10% FBS and treated with 100 μM OA and 100 μM PA for 24 h. The culture medium was replaced with glucose-free DMEM and 100 nM insulin for 10 min, and then the cells were cultured in normal DMEM with 100 μM 2-NBDG for 30 min. The fluorescence intensity at 488 nm was detected by Fluoroskan Ascent FL (Thermo). The cells were plated in 12-well plates, treated with the same methods, and then observed and photographed by fluorescence microscopy (Olympus IX83).

### Immunoblotting

Proteins were extracted quickly on ice using RIPA buffer, and the concentrations were detected with a Bio-Rad protein assay kit (Bio-Rad, USA). A phosphatase inhibitor cocktail was used to extract some protein samples for phosphoprotein detection. The proteins were subjected to 10% SDS‒PAGE and transferred onto PVDF membranes. The membranes were blocked with 5% skimmed milk for 1 h and then incubated overnight at 4 °C with primary antibodies against β-actin (1:100,000, Abclonal), Plin5 (1:1000, homemade), GLUT4 (1:5000, Abclonal), GLUT1 (1:5000, Santa), PGC-1α (1:20,000, Proteintech), PDH (6 µg/ml, Abcam), pPDH (1:10,000, Abclonal), LDHA (1:5000, Abclonal), AKT (1;5000, CST), pAKT S473 (1;5000, CST), CPT1 (1:5000 Abclonal), CPT2 (1:5000 Abclonal), and βMHC (0.4 µg/ml Abcam). After rinsing the PVDF membranes with PBS three times, the membranes were incubated with HRP-conjugated secondary antibodies (Jackson). Finally, the fluorescence signals were detected with an electrochemiluminescence (ECL) detection system (Cytiva Amersham ImageQuant 800). Relative protein expression was normalized to β-actin expression. The band intensity was determined by Image-Pro Plus 6.0 (Media Cybernetic Inc., USA) image analysis software.

### Quantitative PCR

Total RNA was extracted from mouse heart tissues or cells using TRIzol (Invitrogen) according to the manufacturer’s instructions. cDNA was synthesized with an ABScript II cDNA First Strand Synthesis Kit (ABclonal). Quantitative PCR was performed using 2 × Universal SYBR Green Fast qPCR Mix (ABclonal) on an Applied Biosystems StepOne™ Real-Time PCR System Thermal Cycling Block. Each analysis was performed at least three times. Relative gene expression levels were normalized to β-actin expression using the -∆∆CT method.

### Biochemical parameters

The levels of glucose, lactate, NADH and NAD + were measured using commercial assay kits (Beyotime Biotechnology) following the manufacturer’s instructions. The triglyceride (TG) content was measured using a Triglyceride Content Assay Kit (Solarbio Life Science, China).

### Statistical analysis

All data are presented as the mean ± SEM. The statistical significance of the differences between groups was assessed by Student’s* t* test. *P* < 0.05 was considered to indicate statistical significance. Statistical analyses were performed using GraphPad Prism Software.

## Results

### Plin5 deficiency reduced glucose utilization in cardiomyocytes

**Fig. 1:**
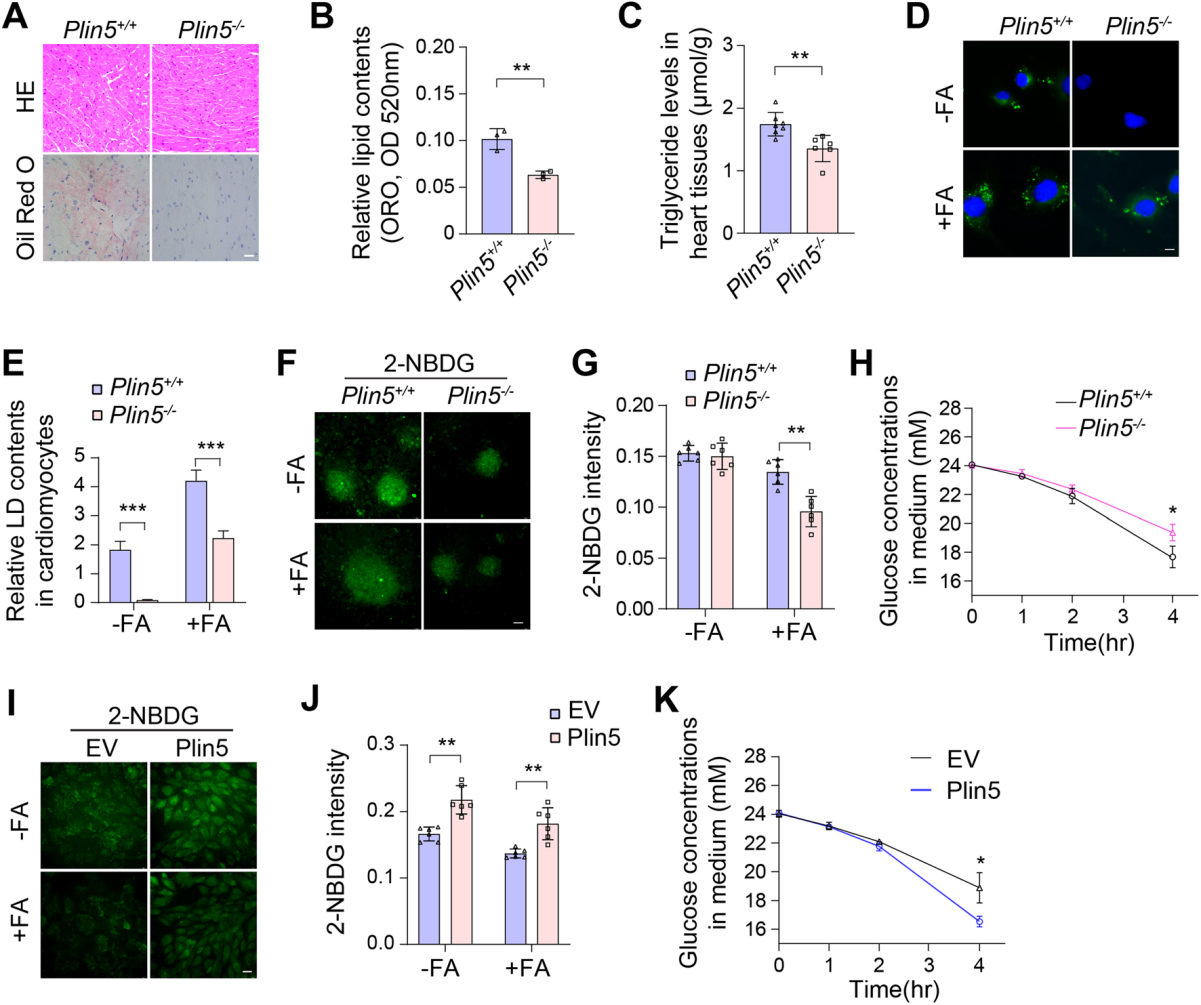
Plin5 deficiency reduced glucose utilization in mouse cardiomyocytes. **A** Representative images of H&E staining and Oil Red O staining of myocardial tissue from wild-type (Plin5^+/+^) and Plin5-knockout (Plin5^−/−^) mice. Scale bar, 50 μm. **B** Quantification analysis of Oil red O staining (n = 3, **P < 0.01). **C** Triglyceride (TG) levels in myocardial tissue of Plin5^+/+^ and Plin5.^−/−^ mice (n = 6, **P = 0.01). **D**, **E** BODIPY 493/503 dye staining in neonatal mouse cardiomyocytes after treatement with FAs for 24 h and quantification analysis. Scale bar, 10 μm. **F**, **G** Fluorescence photomicrograph (**F**) and intensity analysis (**G**) of neonatal mouse cardiomyocytes after 2-NBDG administration for 30 min. Scale bar, 50 μm. (n = 6 in G, **P < 0.01). **H** Neonatal mouse cardiomyocytes were cultured in DMEM with 25 μm glucose and treated with FA for the indicated times, and the glucose concentration in the medium was determined (n = 3, *P < 0.05). **I**, **J** Fluorescence photomicrograph (**I**) and intensity analysis (**J**) of the control (EV) and Plin5-overexpressing (Plin5) H9C2 cells after 2-NBDG administration. Scale bar, 50 μm. (n = 6 in J, **P < 0.01). **K** Glucose concentration in the medium of Plin5-overexpressing H9C2 cells treated with FAs (n = 3, *P < 0.05)

Previous reports have proven that Plin5 preserves lipid droplet (LD) homeostasis by inhibiting lipolysis [15]. In this study, Oil Red O staining showed that the lipid contents were reduced in the heart tissues of Plin5-null mice (Fig. 1A, B). Consistently, a reduction in triglyceride (TG) level was also found in the Plin5-knockout myocardium (Fig. 1C). Then, neonatal mouse cardiomyocytes (CMs) were isolated, and BODIPY 493/503 staining revealed that the content of LDs was significantly reduced in Plin5-null cardiomyocytes as well as in cardiomyocytes treated with FAs (Fig. 1D, E). These results confirmed that Plin5 deficiency elevated lipolysis in cardiomyocytes.

Then, 2-NBDG, a fluorescently labelled form of 2-deoxyglucose, was used as a tracer to investigate the glucose uptake of cardiomyocytes. We found that 2-NBDG uptake was reduced in Plin5-null cardiomyocytes, particularly in the presence of FAs (Fig. 1F). The fluorescence intensity analysis also showed a consistent result (Fig. 1G). In addition, medium glucose consumption analysis indicated that glucose unitization was reduced in Plin5-null cardiomyocytes in the presence of FAs (Fig. 1H). To further explore the roles of Plin5 in the myocardium, Plin5-overexpressing H9C2 cells (Plin5 OE) were generated by lentivirus infection. We found that Plin5 overexpression improved 2-NBDG uptake in H9C2 cells regardless of the presence of FAs (FAs) (Fig. 1I, J). Consistently, medium glucose consumption was also increased in Plin5-overexpressing H9C2 cells treated with FAs (Fig. 1K). Thus, these results implied that Plin5 deficiency reduced glucose utilization in cardiomyocytes, especially in the presence of FAs.

### Plin5 deficiency impaired insulin signalling in cardiomyocytes

**Fig. 2:**
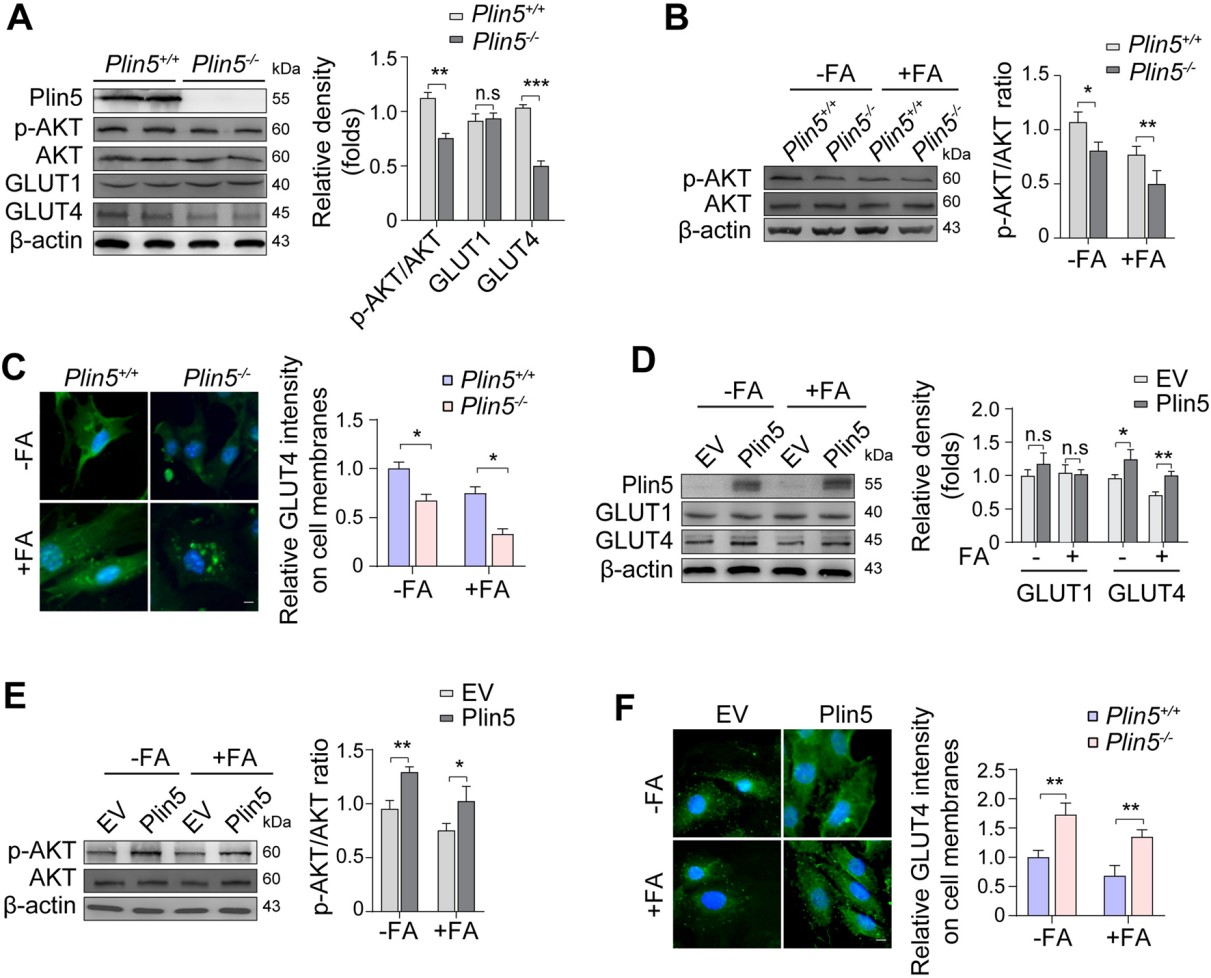
Plin5 deficieny impaired insulin signalling in cardiomyocytes. **A** Expression of AKT, p-AKT, GLUT1, and GLUT4 in wild-type (Plin5^+/+^) and Plin5-knockout (Plin5^−/−^) myocardia (n = 3, **P < 0.01, ***P < 0.001). **B** Expression of AKT and p-AKT in Plin5^+/+^ and Plin5^−/−^ neonatal mouse cardiomyocytes treated with fatty acids (FAs) (n = 3, *P = 0.05, **P = 0.01). **C** Immunofluorescence staining of GLUT4 in Plin5^+/+^ and Plin5^−/−^ neonatal mouse cardiomyocytes and quantification analysis. Scale bar, 10 μm. (n = 3, *P < 0.05). **D** Expression of GLUT1 and GLUT4 in control (EV) and Plin5-overexpressing H9C2 cells (Plin5) (n = 3, *P < 0.05, **P < 0.01). **E** Expression of AKT and p-AKT in control and Plin5-overexpressing H9C2 cells (n = 3, *P < 0.05, **P < 0.01). **F** Immunofluorescence staining of GLUT4 in control and Plin5-overexpressing H9C2 cells and quatification analysis. Scale bar, 10 μm. (n = 3, **P < 0.01)

Both GLUT1 and GLUT4 are the predominant GLUT isoforms expressed in the heart [16], and insulin stimulates GLUT4 translocation to the cardiomyocyte membrane to transport glucose by activating the protein kinase B (PKB)/AKT pathway [16, 17]. As Plin5 deficiency triggered a decrease in glucose uptake in cardiomyocytes, the expression levels of GLUT1 and GLUT4 in the mouse myocardia were determined. We found that there were almost no changes in GLUT1 expression, while a significant decrease in GLUT4 expression was observed in the hearts of Plin5-knockout mice (Fig. 2A). Meanwhile, the phosphorylation (Ser473) of AKT (p-AKT) was significantly reduced in Plin5-deficient heart tissues and neonatal mouse cardiomyocytes, especially in the presence of FAs (Fig. 2A, B). Additionally, immunofluorescence staining revealed that Plin5 deficiency reduced the membrane translocation of GLUT4 in neonatal mouse cardiomyocytes, particularly in the presence of FAs (Fig. 2C). In Plin5-overexpressing H9C2 cells, the expression of GLUT4, but not GLUT1, was upregulated (Fig. 2D). Consistently, Plin5 overexpression increased the p-AKT levels in both the absence and presence of FAs (Fig. 2E). Immunofluorescence staining also revealed that Plin5 overexpression promoted insulin-stimulated membrane translocation of GLUT4 in cardiomyocytes, particularly in the presence of FAs (Fig. 2F). Thus, these results suggested that Plin5 deficiency impaired insulin signalling in cardiomyocytes.

### Plin5 deficiency increased lactate production in cardiomyocytes

**Fig. 3:**
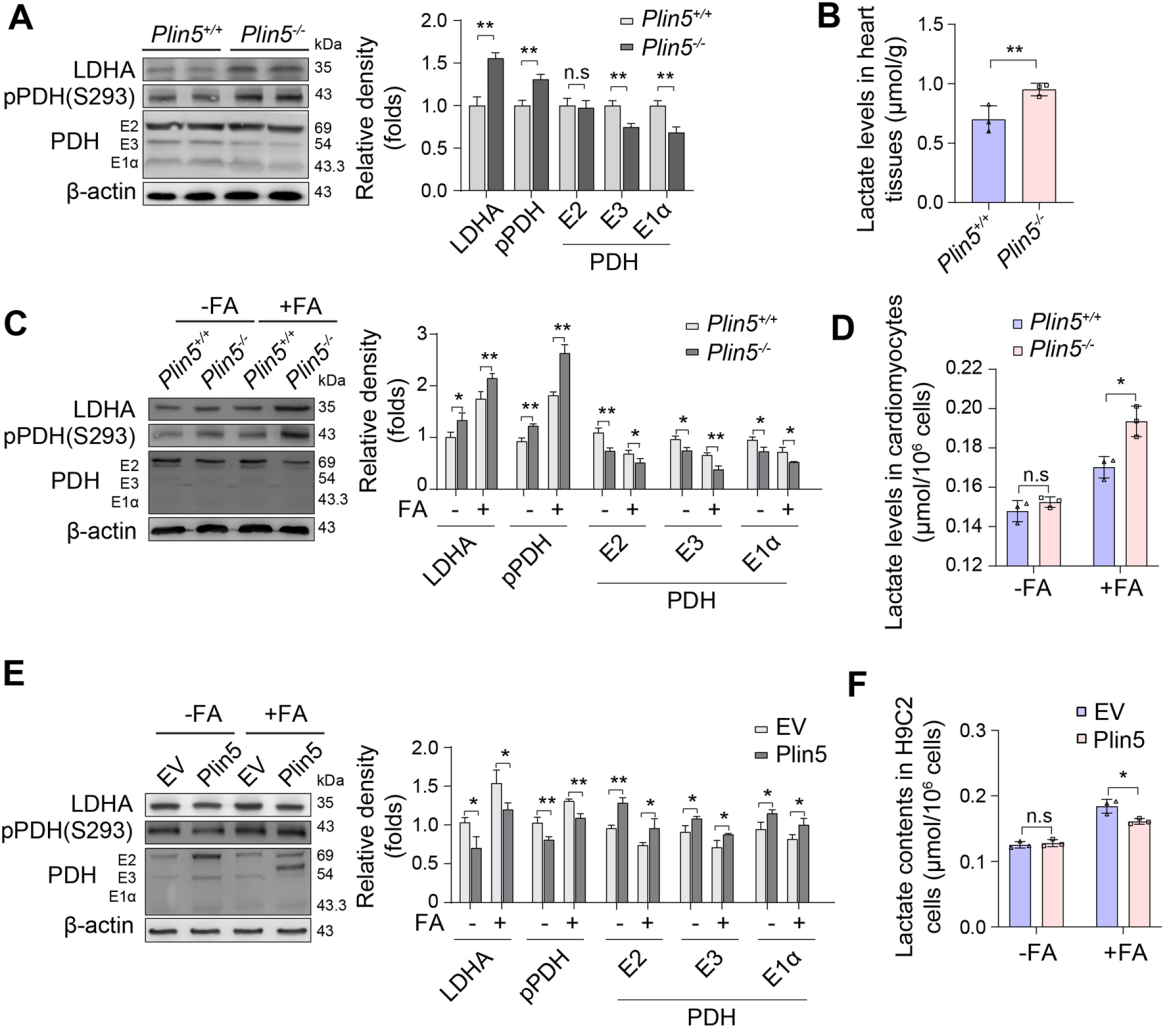
Plin5 deficiency promoted lactate production in cardiomyocytes. **A** The expression levels of PDH, pPDH and LDHA in the myocardia of wild-type (Plin5^+/+^) and Plin5-knockout (Plin5^−/−^) mice were analysed by Western blotting (n = 3, **P < 0.01). **B** Lactate levels in the myocardia of Plin5^+/+^ and Plin5^−/−^ mice (n = 3, **P < 0.05). **C** After treatment with FAs, the expression levels of PDH, pPDH and LDHA were analysed by Western blotting in Plin5^+/+^ and Plin5^−/−^ neonatal mouse cardiomyocytes (n = 3, *P < 0.05, **P < 0.01). **D** The lactate content was also determined (n = 3, *P < 0.05). **E, F** Expression of PDH, pPDH and LDHA (**E**) (n = 3, *P < 0.05, **P < 0.01) and lactate production (**F**) in control (EV) and Plin5-overexpressing (Plin5) H9C2 cells treated with FAs (n = 3, *P < 0.05)

For glucose oxidation, pyruvate is catalysed into acetyl-CoA by pyruvate dehydrogenase (PDH), and acetyl-CoA then enters the TCA cycle. Here, Plin5 knockout caused a decrease in the expression of the pyruvate dehydrogenase (PDH) complex but an increase in the phosphorylation (Ser293) of the E1 subunit (pPDH), which indicated that Plin5 deficiency inactivated PDH activity and reduced glucose oxidation in the mouse heart (Fig. 3A). Interestingly, Plin5 deficiency increased the lactate content (Fig. 3B), and the expression of lactate dehydrogenase (LDHA) was also elevated (Fig. 3A). Consistently, the expression of PDH was downregulated, but the levels of pPDH and LDHA were increased, in the Plin5-null neonatal mouse cardiomyocytes (Fig. 3C). Furthermore, fatty acid exposure increased the lactate content in Plin5-null cardiomyocytes (Fig. 3D). Conversely, Plin5 overexpression increased the expression of PDH in H9C2 cells but decreased the phosphorylation of PDHE1 and the expression of LDHA in the presence of FAs (Fig. 3E). Moreover, Plin5 overexpression attenuated lactate production in H9C2 cells in the presence of FAs (Fig. 3F). Thus, these results indicated that Plin5 deficiency increased lactate production in cardiomyocytes.

### ***Plin5 deficiency decreased the NAD***^+^***/NADH ratio in cardiomyocytes***

**Fig. 4:**
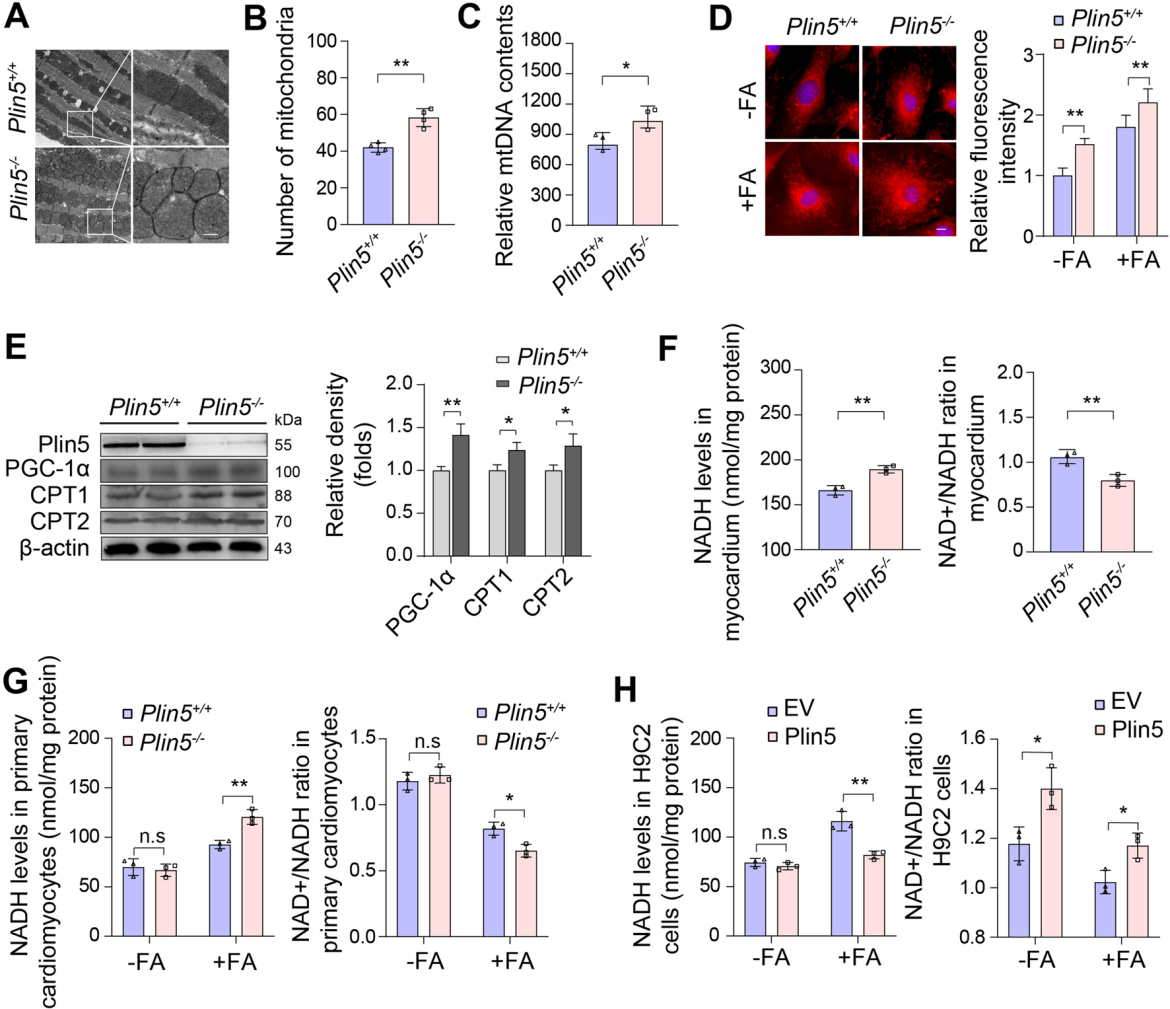
Plin5 deficiency increased NADH levels in cardiomyocytes. **A, B** The myocardia of wild-type (Plin5^+/+^) and Plin5-knockout (Plin5^−/−^) mice were observed by transmission electron microscopy, and the numbers of mitochondria were statistically analysed (n = 3, **P < 0.01). **C** Quantification of mitochondrial DNA in the myocardium of Plin5^+/+^ and Plin5^−/−^ mice (n = 3, *P < 0.05). **D** Representative images and quantification analysis of MitoTracker staining of primary cardiomyocytes from Plin5^+/+^ and Plin5^−/−^ mice with or without FAs) stimulation. Scale bar, 10 μM (n = 3, *P < 0.05, **P < 0.01). **E** Western blotting and quantitive analysis of CPT1, CPT2 and PGC-1α in primary cardiomyocytes from Plin5^+/+^ and Plin5^−/−^ mice (n = 3, *P < 0.05, **P < 0.01). **F** NADH levels and NAD+/NADH ratios in myocardia Plin5^+/+^ and Plin5^−/−^ mice (n = 3, **P < 0.01). **G** NADH levels and NAD+/NADH ratios in primary wild-type (Plin5^+/+^) and Plin5-null (Plin5.^−/−^) cardiomyocytes in the absence and presence of FAs (n = 3, *P < 0.05, **P < 0.01). **H** NADH levels and NAD + /NADH ratios in Plin5-overexpressing H9C2 cells after treatment with or without FAs (n = 3, *P < 0.05, **P < 0.01)

Plin5 plays a very important role in maintaining the stability of intracellular LDs and regulating fatty acid metabolism. Here, our results indicated that the Plin5-null hearts exhibited fewer LDs and increased mitochondria numbers under transmission electron microscopy (Fig. 4A, B) and an increase in mitochondrial DNA according to quantitative PCR analysis (Fig. 4C). Consistently, MitoTracker staining showed that FA treatment stimulated the biogenesis of mitochondria in Plin5-null cardiomyocytes (Fig. 4D). Additionally, PGC-1α, a well-known key regulator of mitochondrial biogenesis, was upregulated in Plin5-null cardiomyocytes (Fig. 4E). Furthermore, Plin5 deficiency increased the expression of both carnitine palmitoyl transferase 1 and carnitine palmitoyl transferase 2 (CPT1 and CPT2), which are located in the outer and inner mitochondrial membrane, respectively, to transport FAs into mitochondria (Fig. 4E). These results indicated an increase in FAO.

As the oxidation of fatty acids can produce NADH, we determined the NADH levels in Plin5-deficient hearts and cardiomyocytes. As expected, Plin5 deficiency increased the NADH content in heart tissues (Fig. 4F). In neonatal cardiomyocytes, Plin5 deficiency significantly elevated the NADH content and reduced the NAD + /NADH ratio, especially in the presence of FAs (Fig. 4G). In contrast, Plin5 overexpression attenuated the FA-induced NADH elevation and increased the NAD+^+^/NADH ratio in H9C2 cells (Fig. 4H). Taken together, the results indicated that Plin5 deficiency increased NADH levels in cardiomyocytes by stimulating excessive FAO.

### Inhibiting lactate production ameliorated insulin resistance in Plin5-deficient cardiomyocytes

**Fig. 5:**
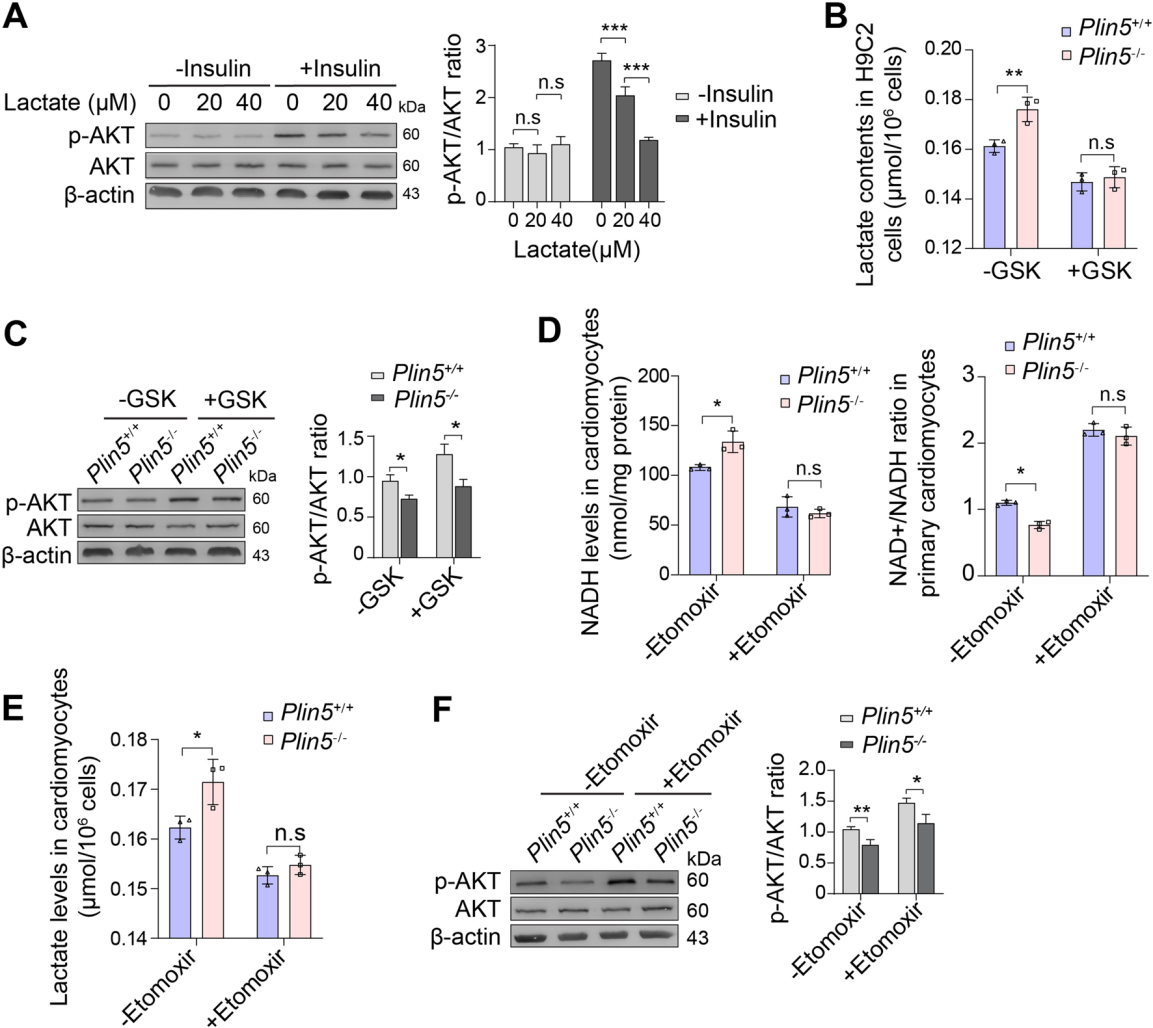
Inhibiting lactate production ameliorated insulin resistance in Plin5-deficient cardiomyocytes. **A** The expression levels of AKT and p-AKT in H9C2 cells were analysed by immunoblotting after treatment with various concentrations of lactate in the absence and presence of insulin (n = 3, ***P < 0.001). **B, C** Wild-type (Plin5^+/+^) and Plin5-null (Plin5^−/−^) CMs were cultured in media supplemented with FAss, lactate levels (**B**) were determined after treatment with 10 μM GSK2837808A, and the expression levels of AKT and p-AKT (**C**) were detected by immunoblotting (n = 3, *P < 0.05). **D–F** The NADH levels, NAD + /NADH ratios (D) and lactate levels (E) were determined in wild-type (Plin5^+/+^) and Plin5-null (Plin5^−/−^) CMs, and the expression of AKT, p-AKT (F) was detected after treatment with 60 μM etomoxir (n = 3, *P < 0.05, **P < 0.01)

Lactate is believed to serve as a multifunctional signalling molecule that mediates many pathophysiological processes. Here, H9C2 cells were treated with various concentrations of lactate, and we found that the insulin-induced phosphorylation of AKT was significantly inhibited (Fig. 5A). In contrast, GSK2837808A, a selective LDHA inhibitor, reduced lactate accumulation (Fig. 5B) and then increased the phosphorylation of AKT in Plin5-deficient cardiomyocytes (Fig. 5C). These results indicated that lactate accumulation in Plin5-deficient cardiomyocytes impaired insulin signalling. As lactate accumulation in Plin5-deficient cardiomyocytes was caused by the elevation in NADH from excessive FAO, we inhibited FAO with etomoxir, an irreversible CPT1 inhibitor. Our results showed that the inhibition of FAO attenuated the elevations in both NADH and lactate in Plin5-null mouse cardiomyocytes, even in the presence of FAs (Fig. 5D, E). More importantly, fatty acid inhibition also improved the insulin-induced phosphorylation of AKT in Plin5-null mouse cardiomyocytes (Fig. 5F). These results confirmed that lactate accumulation derived from excessive FAO impaired insulin signalling in Plin5-deficient cardiomyocytes.

### Plin5 deficiency aggravated cardiac hypertrophy in leptin-deficient mice

**Fig. 6:**
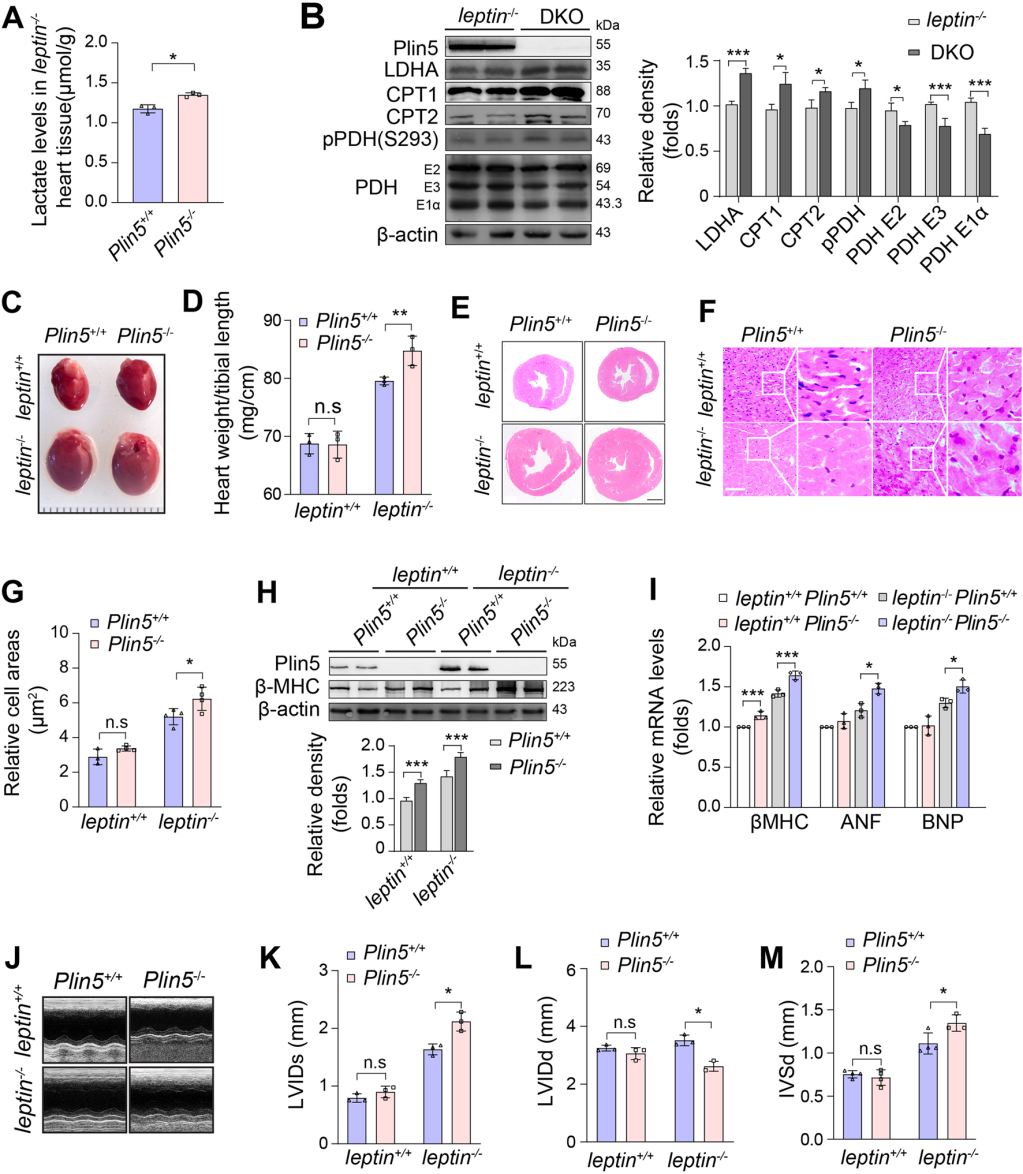
Plin5 deficiency exacerbated cardiac hypertrophy in leptin-deficient mice. **A**, **B** Lactate levels (**A**) and the expression of LDHA, CPT1, CPT2, PDH, pPDH (**B**) in the myocardial tissues of leptin-deficient (Plin5^+/+^/leptin^−/−^) and Plin5/leptin-double-knockout (DKO) mice (n = 3, *P < 0.05, ***P < 0.001). **C**–**F** Gross and histological features of the cardiomyocytes in Plin5/leptin single or double-knockout mice; a cross-section of the heart was taken for morphological observation. Scale bar, 50 μM. **G** Statistical analysis of the cross-sectional area of myocardium from Plin5/leptin single or double-knockout (DKO) mice (n = 4, *P < 0.01). **H** Expression of βMHC, in the myocardial tissues of Plin5/leptin-double-knockout mice (n = 3, ***P < 0.001). **I** mRNA levels of βMHC, ANF, and BNP in the Myocardial tissues of Plin5- and leptin-DKO mice as determined by qPCR. **J**–**M** Effect of Plin5 knockout on end-systolic variable in Plin5/leptin-knockout (Plin5^+/+^ or leptin^−/−^) or DKO mice

Lactate accumulation is involved in myocardial and skeletal hypertrophic growth [18, 19], but we did not find obvious cardiac hypertrophy in Plin5-deficient mice. As leptin-deficient mice are characterized by abnormal lipid metabolism and cardiac hypertrophy, we generated Plin5/leptin-double-knockout (DKO) mice and found that lactate levels were significantly elevated in the myocardia of Plin5/leptin-DKO mice compared with those of leptin-knockout mice (Fig. 6A). Moreover, immunoblotting showed that Plin5 deficiency significantly elevated the expression of CPT1, CPT2, pPDH and LDHA but decreased the expression of PDH in the myocardium in leptin-deficient mice (Fig. 6B). Importantly, we found that the heart volumes were enlarged and that the ratio of heart weight to tibia length was increased in Plin5/leptin-DKO mice compared with leptin-deficient mice, but there were no significant differences between wild-type and Plin5-knockout mice (Fig. 6C, D). In addition, H&E staining showed that Plin5 deficiency increased the thickness of the ventricular wall and septum only in leptin-deficient mice (Fig. 6E, F). Statistically, Plin5/leptin-DKO mice exhibited a 1.5-fold greater mean diameter of cardiomyocytes than leptin-knockout mice (Fig. 6G). Furthermore, the mRNA levels of cardiac hypertrophy markers, such as ANF, BNF and β-MHC, were significantly upregulated in the myocardium in Plin5/leptin-DKO mice compared with Plin5-wild-type and leptin-deficient mice. (Fig. 6H, I). Consistently, the cardiac ultrasound parameters also showed that Plin5 deficiency impaired diastolic functions in leptin-deficient mice by affecting variables including the left ventricular end-systolic internal dimension (LVIDs), left ventricular end-diastolic internal dimension (LVIDd), and interventricular septum thickness in diastole (IVSd) (Fig. 6J, M). These data indicated that Plin5 deficiency aggravated cardiac hypertrophy in leptin-deficient mice.

## Discussion

Plin5 has emerged as an essential regulator of lipolysis in oxidative tissues that functions by maintaining the stability of LDs [20] and has been reported to regulate mitochondrial proliferation and oxidative activity [21, 22]. Plin5 anchors mitochondria to the LD membrane via its last 20 C-terminal amino acids, which is considered to augment respiratory capacity [23]. It seems to be required for adaptation to lipid overload and protects mitochondria against excessive free fatty acid inflow. Plin5 overexpression promotes LD formation and mitochondria–LD contact, reduces cellular ROS levels and upregulates mitochondrial function-related genes [24]. Plin5 is enriched in the heart, which utilizes large amounts of FAs as energy-providing substrates. Existing research suggests that Plin5 can prevent type 1 diabetes-induced heart malfunction [25] and protect the heart from ischaemia‒reperfusion injury by inhibiting the lipolysis of LDs [13, 26]. Conversely, cardiac-specific overexpression of Plin5 provokes cardiac steatosis via the formation of a lipolytic barrier [27, 28]. However, the roles of Plin5 in myocardial diseases need to be intensively investigated.

Our previous study showed that Plin5 deficiency exacerbated pressure overload-induced cardiac hypertrophy and heart failure by enhancing myocardial FAO and oxidative stress [11]. In this study, we found that mitochondria numbers were significantly increased in Plin5-deficient hearts and that these increases were accompanied by excessive FAO in mitochondria. More importantly, we found that lactate accumulated in Plin5-deficient cardiomyocytes due to the elevation in NADH. Moreover, we found that Plin5 deficiency-induced lactate accumulation aggravated myocardial hypertrophy in leptin-deficient mice. These results suggested that Plin5 could control the balance of FAO and glucose oxidation in cardiomyocytes, which is very important for maintaining the structure and function of the heart.

Except for these basic roles in controlling lipid metabolism, the regulation of glucose metabolism in cardiomyocytes by Plin5 has been poorly clarified. We observed that glucose uptake was significantly reduced in Plin5-deficient myocardia but that lactate production was increased, especially in the presence of FAs or in leptin-deficient mice. Moreover, lactate accumulation impaired insulin signalling and inhibited glucose uptake by reducing the phosphorylation of AKT in Plin5-deficient cardiomyocytes. Recent studies have shown that lactate is the key trigger that mediates obesity-induced inflammation and systemic insulin resistance [29]. Human adipose lactate levels are positively linked with local inflammatory features and the insulin resistance index independent of body mass index (BMI) [30]. Our results indicated that Plin5 deficiency promoted anaerobic glycolysis, resulting in insulin resistance in cardiomyocytes.

Excessive FAO can increase the production of acetyl-coenzyme A (acetyl-CoA) and reductive NADH that activate pyruvate dehydrogenase kinase 4 (PDK4), which inhibits glucose utilization by inactivating PDH [31–33]. Our results showed that NADH levels were significantly elevated in Plin5-null myocardia. Excessive NADH not only promoted the reduction of pyruvate into lactate but also inhibited PDH activity, resulting in decreased glucose oxidation. Inhibition of FAO by etomoxir attenuated the NADH levels in cardiomyocytes, ameliorated lactate production and improved insulin signalling. In addition to NADH, acetyl-CoA produced by FAO has been reported to increase global protein acetylation and to inhibit glucose uptake in cardiomyocytes [34]. In further studies, the regulation of oxidative metabolism by protein acetylation should be intensively investigated in Plin5-deficient cardiomyocytes.

Leptin-deficient mice exhibit myocardial hypertrophy, which is closely associated with insulin resistance, altered substrate utilization, mitochondrial dysfunction, and lipid accumulation. Insulin resistance and increased fatty acid utilization are major metabolic features of obesity and the diabetic myocardium [35]. In diabetes, various types of lipotoxic intermediates produced by FA overoxidation are involved in the development of myocardial hypertrophy [35–37]. In this study, no obvious morphological changes were observed in the hearts between Plin5-deficient and wild-type mice, but Plin5 deficiency significantly exacerbated myocardial hypertrophy. Lactate is believed to serve as a multifunctional signalling molecule that mediates many pathophysiological processes, and a recent study showed that lactate is necessary in myocardial hypertrophy [18]. Inhibition of the pyruvate transporter (MPC) directing glucose towards lactate can drive cardiomyocyte hypertrophic growth, but inhibition of the lactate transporter (MCT4) directing glucose towards pyruvate inhibits cardiomyocyte hypertrophic growth [19]. In this study, we found that lactate accumulation due to Plin5 deficiency aggravated myocardial hypertrophy in leptin-deficient mice. Thus, lactate acts as a new important messenger metabolite to stimulate myocardial hypertrophy in Plin5-deficient mice.

In summary, this study confirmed that Plin5 deficiency promoted lactate accumulation due to excessive NADH production from FAO in cardiomyocytes, which impaired insulin signalling and resulted in myocardial hypertrophy in leptin-deficient mice. These results imply that Plin5 orchestrates the balance of fatty acid and glucose oxidation and protects cardiomyocytes against fatty acid overload. Our data also provide evidence that Plin5 is a new target for preventing and delaying the onset of myocardial hypertrophy in obesity and diabetes mellitus.

## Conclusion

These results indicate that excessive FAO in Plin5-deficient cardiomyocytes can stimulate lactate production by increasing NADH levels. The increased lactate production by Plin5 deficiency plays a critical role in myocardial substrate metabolism disorders. Collectively, our results demonstrate that Plin5 is highly related to the coordination of fatty acid and glucose oxidation in cardiomyocytes, providing a potential target for the treatment of metabolic disorders in the heart.

## Data Availability

The datasets used and/or analysed during the current study are available from the corresponding author upon reasonable request.

## Abbreviations

Plin5: Perilipin 5
LDs: Lipid droplets
LDHA: Lactate dehydrogenase
FAO: Fatty acid β-oxidation
BAT: Brown adipose tissue
TG: Triglyceride
CMs: Neonatal mouse cardiomyocytes
Plin5 OE: Plin5-overexpressing H9C2
PDH: Pyruvate dehydrogenase
pPDH: Phosphorylated (Ser293) E1 subunit of PDH
CPT1/2: Carnitine palmitoyl transferase 1/2
DKO: Double-knockout
LVIDs: Left ventricular end-systolic internal dimension
LVIDd: Left ventricular end-diastolic internal dimension
IVSd: Interventricular septum thickness in diastole
BMI: Body mass index
PDK4: Pyruvate dehydrogenase kinase 4
MPC: Pyruvate transporter
MCT4: Lactate transporter
FAs: Fatty acids

## References

[1] Ritterhoff J, Tian R (2017). Metabolism in cardiomyopathy: every substrate matters. Cardiovasc Res.

[2] Lopaschuk GD, Jaswal JS (2010). Energy metabolic phenotype of the cardiomyocyte during development, differentiation, and postnatal maturation. J Cardiovasc Pharmacol.

[3] Stanley WC (2004). Myocardial energy metabolism during ischemia and the mechanisms of metabolic therapies. J Cardiovasc Pharmacol Ther.

[4] Kolwicz SC, Purohit S, Tian R (2013). Cardiac metabolism and its interactions with contraction, growth, and survival of cardiomyocytes. Circ Res.

[5] Turer AT (2013). Using metabolomics to assess myocardial metabolism and energetics in heart failure. J Mol Cell Cardiol.

[6] Bayeva M, Sawicki KT, Ardehali H (2013). Taking diabetes to heart–deregulation of myocardial lipid metabolism in diabetic cardiomyopathy. J Am Heart Assoc.

[7] Yan D, Cai Y, Luo J, Liu J, Li X, Ying F, Xie X, Xu A, Ma X, Xia Z (2020). FOXO1 contributes to diabetic cardiomyopathy via inducing imbalanced oxidative metabolism in type 1 diabetes. J Cell Mol Med.

[8] Tran DH, Wang ZV (2019). Glucose metabolism in cardiac hypertrophy and heart failure. J Am Heart Assoc.

[9] Carley AN, Bi J, Wang X, Banke NH, Dyck JR, O'Donnell JM, Lewandowski ED (2013). Multiphasic triacylglycerol dynamics in the intact heart during acute in vivo overexpression of CD36. J Lipid Res.

[10] Kienesberger PC, Pulinilkunnil T, Nagendran J, Dyck JR (2013). Myocardial triacylglycerol metabolism. J Mol Cell Cardiol.

[11] Wang C, Yuan Y, Wu J, Zhao Y, Gao X, Chen Y, Sun C, Xiao L, Zheng P, Hu P (2019). Plin5 deficiency exacerbates pressure overload-induced cardiac hypertrophy and heart failure by enhancing myocardial fatty acid oxidation and oxidative stress. Free Radic Biol Med.

[12] Kuramoto K, Okamura T, Yamaguchi T, Nakamura TY, Wakabayashi S, Morinaga H, Nomura M, Yanase T, Otsu K, Usuda N (2012). Perilipin 5, a lipid droplet-binding protein, protects heart from oxidative burden by sequestering fatty acid from excessive oxidation. J Biol Chem.

[13] Mason RR, Mokhtar R, Matzaris M, Selathurai A, Kowalski GM, Mokbel N, Meikle PJ, Bruce CR, Watt MJ (2014). PLIN5 deletion remodels intracellular lipid composition and causes insulin resistance in muscle. Mol Metab.

[14] Keenan SN, Meex RC, Lo JCY, Ryan A, Nie S, Montgomery MK, Watt MJ (2019). Perilipin 5 deletion in hepatocytes remodels lipid metabolism and causes hepatic insulin resistance in mice. Diabetes.

[15] Wang C, Zhao Y, Gao X, Li L, Yuan Y, Liu F, Zhang L, Wu J, Hu P, Zhang X (2015). Perilipin 5 improves hepatic lipotoxicity by inhibiting lipolysis. Hepatology.

[16] Bertrand L, Auquier J, Renguet E, Ange M, Cumps J, Horman S, Beauloye C (2020). Glucose transporters in cardiovascular system in health and disease. Pflugers Arch.

[17] Petersen MC, Shulman GI (2018). Mechanisms of insulin action and insulin resistance. Physiol Rev.

[18] Dai C, Li Q, May HI, Li C, Zhang G, Sharma G, Sherry AD, Malloy CR, Khemtong C, Zhang Y (2020). Lactate dehydrogenase a governs cardiac hypertrophic growth in response to hemodynamic stress. Cell Rep.

[19] Cluntun AA, Badolia R, Lettlova S, Parnell KM, Shankar TS, Diakos NA, Olson KA, Taleb I, Tatum SM, Berg JA (2021). The pyruvate-lactate axis modulates cardiac hypertrophy and heart failure. Cell Metab.

[20] Wang H, Bell M, Sreenivasan U, Sreenevasan U, Hu H, Liu J, Dalen K, Londos C, Yamaguchi T, Rizzo MA (2011). Unique regulation of adipose triglyceride lipase (ATGL) by perilipin 5, a lipid droplet-associated protein. J Biol Chem.

[21] Gallardo-Montejano VI, Saxena G, Kusminski CM, Yang C, McAfee JL, Hahner L, Hoch K, Dubinsky W, Narkar VA, Bickel PE (2016). Nuclear Perilipin 5 integrates lipid droplet lipolysis with PGC-1alpha/SIRT1-dependent transcriptional regulation of mitochondrial function. Nat Commun.

[22] Najt CP, Khan SA, Heden TD, Witthuhn BA, Perez M, Heier JL, Mead LE, Franklin MP, Karanja KK, Graham MJ (2020). Lipid droplet-derived monounsaturated fatty acids traffic via PLIN5 to allosterically activate SIRT1. Mol Cell.

[23] Kien B, Kolleritsch S, Kunowska N, Heier C, Chalhoub G, Tilp A, Wolinski H, Stelzl U, Haemmerle G (2022). Lipid droplet-mitochondria coupling via perilipin 5 augments respiratory capacity but is dispensable for FA oxidation. J Lipid Res.

[24] Tan Y, Jin Y, Wang Q, Huang J, Wu X, Ren Z (2019). Perilipin 5 protects against cellular oxidative stress by enhancing mitochondrial function in HepG2 cells. Cells.

[25] Kuramoto K, Sakai F, Yoshinori N, Nakamura TY, Wakabayashi S, Kojidani T, Haraguchi T, Hirose F, Osumi T (2014). Deficiency of a lipid droplet protein, perilipin 5, suppresses myocardial lipid accumulation, thereby preventing type 1 diabetes-induced heart malfunction. Mol Cell Biol.

[26] Zheng P, Xie Z, Yuan Y, Sui W, Wang C, Gao X, Zhao Y, Zhang F, Gu Y, Hu P (2017). Plin5 alleviates myocardial ischaemia/reperfusion injury by reducing oxidative stress through inhibiting the lipolysis of lipid droplets. Sci Rep.

[27] Wang H, Sreenivasan U, Gong DW, O'Connell KA, Dabkowski ER, Hecker PA, Ionica N, Konig M, Mahurkar A, Sun Y (2013). Cardiomyocyte-specific perilipin 5 overexpression leads to myocardial steatosis and modest cardiac dysfunction. J Lipid Res.

[28] Pollak NM, Schweiger M, Jaeger D, Kolb D, Kumari M, Schreiber R, Kolleritsch S, Markolin P, Grabner GF, Heier C (2013). Cardiac-specific overexpression of perilipin 5 provokes severe cardiac steatosis via the formation of a lipolytic barrier. J Lipid Res.

[29] Lin Y, Bai M, Wang S, Chen L, Li Z, Li C, Cao P, Chen Y (2022). Lactate Is a key mediator that links obesity to insulin resistance via modulating cytokine production from adipose tissue. Diabetes.

[30] Feng T, Zhao X, Gu P, Yang W, Wang C, Guo Q, Long Q, Liu Q, Cheng Y, Li J (2022). Adipocyte-derived lactate is a signalling metabolite that potentiates adipose macrophage inflammation via targeting PHD2. Nat Commun.

[31] Hue L, Taegtmeyer H (2009). The Randle cycle revisited: a new head for an old hat. Am J Physiol Endocrinol Metab.

[32] Lucchinetti E, Lou PH, Hersberger M, Clanachan AS, Zaugg M (2020). Diabetic rat hearts show more favorable metabolic adaptation to omegaven containing high amounts of n3 fatty acids than intralipid containing n6 fatty acids. Anesth Analg.

[33] Jelinek BA, Moxley MA (2021). Detailed evaluation of pyruvate dehydrogenase complex inhibition in simulated exercise conditions. Biophys J.

[34] De Loof M, Renguet E, Ginion A, Bouzin C, Horman S, Beauloye C, Bertrand L, Bultot L (2023). Enhanced protein acetylation initiates fatty acid-mediated inhibition of cardiac glucose transport. Am J Physiol Heart Circ Physiol.

[35] Fukushima A, Lopaschuk GD (2016). Acetylation control of cardiac fatty acid β-oxidation and energy metabolism in obesity, diabetes, and heart failure. Biochim Biophys Acta.

[36] Chen X, Zhang L, He H, Sun Y, Shen Q, Shi L (2020). Increased O-GlcNAcylation induces myocardial hypertrophy. In Vitro Cell Dev Biol Anim.

[37] Cibi DM, Sandireddy R, Bogireddi H, Tee N, Ghani S, Singh BK, Mackman N, Singh MK, Singh A (2021). Cardiac tissue factor regulates inflammation, hypertrophy, and heart failure in mouse model of type 1 diabetes. Diabetes.

